# Fusion of Intraoperative Transrectal Ultrasound Images with Post-implant Computed Tomography and Magnetic Resonance Imaging

**DOI:** 10.7759/cureus.2394

**Published:** 2018-03-29

**Authors:** Guila Delouya, Jean-Francois Carrier, Renée Xavier-Larouche, Yannick Hervieux, Dominic Béliveau-Nadeau, David Donath, Daniel Taussky

**Affiliations:** 1 Department of Radiation Oncology, Centre hospitalier de l’Université de Montréal (CHUM); 2 Department of Radiation Oncology, Centre hospitalier de l'Université de Montréal (CHUM)

**Keywords:** permanent brachytherapy, dosimetry, mri

## Abstract

Purpose

To compare the impact of the fusion of intraoperative transrectal ultrasound (TRUS) images with day 30 computed tomography (CT) and magnetic resonance imaging (MRI) on prostate volume and dosimetry.

Methods and materials

Seventy-five consecutive patients with CT and MRI obtained on day 30 with a Fast Spin Echo T2-weighted magnetic resonance (MR) sequence were analyzed. A rigid manual registration was performed between the intraoperative TRUS and day-30 CT based on the prostate volume. A second manual rigid registration was performed between the intraoperative TRUS and the day-30 MRI. The prostate contours were manually modified on CT and MRI. The difference in prostate volume and dosimetry between CT and MRI were compared.

Results

Prostate volume was on average 8% (standard deviation (SD) ± 16%) larger on intraoperative TRUS than on CT and 6% (18%) larger than on MRI. In 48% of the cases, the difference in volume on CT was > 10% compared to MRI. The difference in prostate volume between CT and MRI was inversely correlated to the difference in D90 (minimum dose that covers 90% of the prostate volume) between CT and MRI (r = -0.58, P < .001). A D90 < 90% was found in 5% (n = 4) on MRI and in 10% (n = 7) on CT (Fisher exact test one-sided P = .59), but in no patient was the D90 < 90% on both MRI and CT.

Conclusions

When fusing TRUS images with CT and MRI, the differences in prostate volume between those modalities remain clinically important in nearly half of the patients, and this has a direct influence on how implant quality is evaluated.

## Introduction

As a modality of the evaluation of implant quality, magnetic resonance imaging (MRI) is becoming more and more widespread because of its superior ability to delineate the prostate from its surrounding tissue [1]. To evaluate the quality of the implant, dosimetry is done on a computed tomography (CT) scan performed 30 days (± seven days) following the procedure (day 30). An evaluation of dosimetry on CT is complicated because of the artifacts caused by the seeds, thereby triggering difficulties in delineating prostate borders. On the other hand, it seems impractical to use scarce medical resources to determine the quality of an implant, especially because it is done post-facto and since most published data on implant dosimetry and its correlation with clinical outcome have been done on CT [2].

Furthermore, it was our impression, after contouring on post-implant CTs for many years with residents and fellows, that a good implant does not depend so much on the skill of the person contouring. The contouring had little bearing on whether an implant was a good implant.

Similarly, it has been shown that implants considered as excellent quality on the day of the implant were not influenced by the degree of edema subsequently on days eight and 30 [3].

This present study aims to evaluate whether the fusion of images from an intraoperative trans-rectal ultrasound (TRUS) with MRI can improve the accuracy of prostate volume determination in postimplant dosimetry on day 30.

## Materials and methods

Seventy-five consecutive patients treated with I-125 permanent seed prostate brachytherapy (PB) from November 2013 to September 2014 were evaluated, of which six received 110 Gy as a brachytherapy boost to the external beam radiation therapy received. All the other patients received the standard 144 Gy dose.

Patients were treated with the FIRST system (Nucletron, an Elektra Company, Columbia, MD, USA) using TRUS-guided intraoperative interactive planning. For TRUS, the Flex Focus 400 (BK Medical, Peabody, MA, US) was used. The intraoperative prostate volume was contoured on 2.5-mm thick slices. To determine the prostate volume on TRUS, a three-dimensional TRUS acquisition in the treatment position was performed with two fixation needles inserted into the prostate. These needles have a special harpoon to avoid prostate movements as much as possible. The prostate volume was then contoured by one of the two treating radiation oncologists in the operating room.

On day 30 after implant, a CT and MRI were done. The CT scan was performed with 3-mm thick slices. The Fast Spin-Echo T2-weighted 1.5 T MR sequence was used according to Crook et al. [4].

First, a rigid manual registration was performed between the intraoperative TRUS and day-30 CT based on the prostate volume. Then, the prostate contours—originally drawn on the intraoperative TRUS—were modified to adjust to the CT images. Second, another rigid manual registration was performed between the intraoperative TRUS and the day-30 MRI based on the prostate volume. The prostate contours were once again modified to adjust to the MRI images. Seed positions were reconstructed on the CT images. Post-implant dosimetry was performed twice with two different contour sets: day-30 CT and day-30 MRI.

A statistical analysis was done using SPSS 17.0 for Windows (IBM SPSS, Chicago, Illinois). Differences between groups were calculated using the student t-test. A difference of < .05 was considered statistically significant.

## Results

Differences in prostate volume between imaging modalities

Prostate volume was on average 8% (standard deviation (SD), 16%) larger on intraoperative TRUS than on CT and 6% ± 18% (mean ± SD) larger than on MRI. The difference for all patients between CT and MRI was 1.4% ± 18%. However, the difference between CT and MRI was larger than 10% in 48% of patients. A difference greater than 10% was found between TRUS and CT in 35% and between TRUS and MRI in 49% of patients (see Table 1 for more details). The mean difference in cm^3^ was relatively small: TRUS-CT, 2.4 ± 5.1 cm^3^; TRUS-MRI, 1.2 ± 6.0 cm^3^; and CT-MRI, 1.4 ± 6.2 cm^3^.

**Table 1:** Differences in prostate volume after fusion between intraoperative transrectal ultrasound (TRUS) images with post-implant CT and with post-implant MRI on day 30. All differences in prostate volume between each other are P <.001 (student’s t-test). Abbreviations: Intraop, Intraoperative; SD, standard deviation; CT, computed tomography; MRI, magnetic resonance imaging; TRUS, transrectal ultrasound.

	Intraop. Vol.	CT Vol.	MRI Vol.	Δ Vol. TRUS/CT (%)	Δ Vol. TRUS/MRI (%)	Δ Vol. CT/MRI (%)
Mean	34.9	32.1	33.8	108.1	105.9	98.6
SD	12.1	10.3	12.7	16	18	18
Median	34	31	31	107	101	98
Minimum	16	16	16	87	7	51
Maximum	81	67	73	155	160	151

Influence of difference in prostate volume on dosimetry

We then analyzed whether this small difference in prostate volume between CT and MRI would translate into a difference in prostate dosimetry. The mean difference in V100, V150, and D90 were -1.1% ± 11.2%, 2.4% ± 7.0%, and 3.8 ± 23 Gy (Table 2). Although the mean difference for all patients calculated together was small, the difference between CT and MRI was often clinically significant. There was a difference greater than 5% for 33% of cases in V100 and for 41% in V150. A difference greater than 5 Gy for the D90 was found in 72% of cases.

**Table 2:** Differences in prostate dosimetry between post-implant CT and with post-implant MRI on day 30 All differences in dosimetry between each other are P <.001 (student’s t-test). Abbreviations: SD, standard deviation; CT, computed tomography; MRI, magnetic resonance imaging; TRUS, transrectal ultrasound; D90, minimum dose that covers 90% of the prostate volume; V100, prostate volume receiving 100% of the prescribed dose (in %); V150, prostate volume receiving 150% of the prescribed dose (in %).

	D90 on CT	D90 on IRM	ΔD90 CT/MRI (Gy)	V100 on CT	V100 on MRI	ΔV100 CT/MRI (%)	V150 on CT	V150 on MRI	Δ V150 CT/MRI (%)
Mean	158.0	154.1	3.8	91.2	92.3	-1.1	61.5	58.8	2.4
SD	25.1	19.4	23	10.4	4.7	11	13.3	12.1	7
Median	163	155	2	95	93	0	63	59	2
Minimum	93	97	-62	47	71	-52	32	29	-10
Maximum	206	208	50	99	99	20	95	87	39

**Figure 1:**
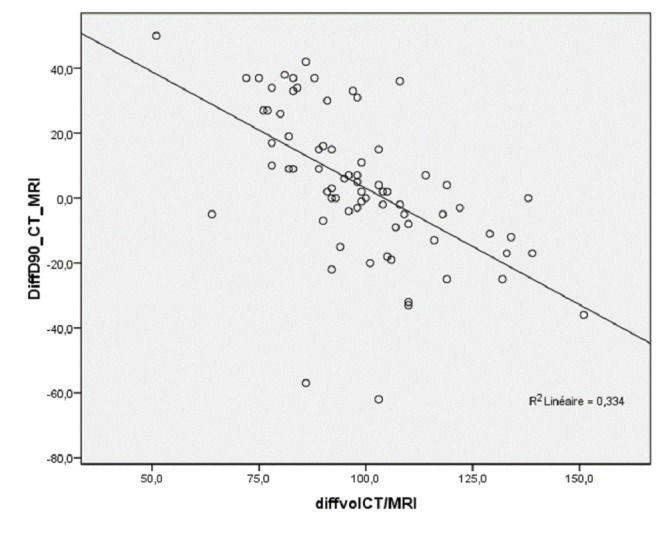
Correlation between the difference in prostate volume between CT and MRI (in %) on day 30 and the difference in D90 between CT and MRI (in Gy) on day 30. Abbreviations: CT, computed tomography; MRI, magnetic resonance imaging.

The fact that the difference in prostate volume between imaging modalities had a direct influence on D90 is best illustrated in Figure 1: The difference in prostate volume was inversely correlated to the difference in D90 between CT and MRI (r=-0.58, p<0.001).

A D90 < 130 Gy was found in 5% (n = 4) of patients on MRI and in 10% (n = 7) on CT (Fisher’s exact test one-sided P = .59). None of the patients had D90 < 130 Gy on both MRI and CT. V100 was ≤ 90% in 21% (n = 16) on CT and 20% (n = 15) on MRI (P = .18). The V100 was calculated as < 90% on both MRI and CT in five patients.

## Discussion

Whether an implant can be labeled “good” depends considerably on the imaging modality used for dosimetry evaluation. In our present study, only in a minority of patients did both dosimetry on MRI and CT agree on whether D90 was > 130 Gy or V100 > 90%.

To our knowledge, we are the first group to copy contours from the prostate on intraoperative TRUS and merge them with the CT and MRI on day 30 and, thus, to assure consistency in prostate volume definition. Superficially, this seemed to work well with an average difference in prostate volume between TRUS and CT of only 8.1% and between TRUS and MRI of 5.9%. The mean difference between CT and MRI was even smaller, with a difference of 1.4%. The large standard deviations of differences in prostate volume between imaging modalities in 35%-49% of patients where the difference was larger than +-10% were surprising to us and underline the inherent difficulties in evaluating postoperative dosimetry: reporting implant quality depends very much on imaging modality and the observer. Unfortunately, we did not perform an interobserver variability analysis that could have shown which imaging modality has less interobserver variability.

We were able to show that this difference in prostate volume matters and why an exact definition of the prostate volume is so important; the difference in prostate volume between CT and MRI was significantly inversely correlated to D90 (r = -0.58, P < .001). Our study contributes to the ongoing debate on whether D90 is predictive of biochemical outcome [5-6]. We were able to show, not surprisingly, that despite great efforts to identify the prostate correctly with the help of adding the intraoperative TRUS, D90 was still very variable. Therefore, our study underlines the need for each center to evaluate their way of contouring the prostate on their postoperative CT or MRI and analyze whether dosimetry in their center predicts outcomes.

In a very interesting and similar paper, Bowes et al. [7] compared TRUS-CT fusion with CT-MRI fusion in 20 patients. Their study differentiates from ours in that CT and MRI were manually fused on seed positions, while we merged on the prostate itself. Bowes et al. fused the TRUS-CT images on the urethral position overlying sagittal positions [7]. We believe that their CT-MRI fusion on the seed position is advantageous compared to our technique, but such a fusion was not technically possible with our software. On the other hand, we believe that their TRUS-CT fusion was less optimal because the urethra position and curvature can change significantly with the catheter position. They found that six of 20 patients had a difference of at least 5% in D90 between MRI and TRUS. Contrary to our findings, a difference in V100 of > 5% was present in only one patient.

It has been recognized that there is significant variability in contouring structures, even among experts in the field. Therefore, there are efforts to develop tools to account for contouring uncertainty, to quantify experts’ level of agreement, and to estimate a consensus structure [8]. MRI, especially on 3.0-Tesla, has been shown to have less interobserver variability than contouring on CT, especially at the level of the prostate base [9]. Automated atlas-based segmentation algorithms have been created to aid in the delineation of target volumes. An “atlas” of expertly contoured CT images for different organs has been created as a model and guide for target volumes' delineation. In one study, there was a moderate agreement (dice similarity coefficient (DSC) 0.71) for prostate contour values contoured by an expert genitourinary radiation oncologist compared to CT atlases [10]. It has been long recognized that prostate volume estimation on MRI and CT is very observer dependent, as is its influence on prostate dosimetry [11].

Crook et al. analyzed 10 patients treated with PB. Prostate contouring was done on MRI and CT [4]. They found an average variation in the prostate volume of 4.1 to 5.9 cubic centimeters (cc). Interestingly, similarly to the results of our study, they found a small mean difference in prostate volume.

The principal investigator who had the preplan TRUS at his disposition found a 2.6 cc (SD, 1.6 cc) difference between preplan TRUS and CT at one month. Again, similar to our study, the impact of contours on dosimetry was considerable; V100 on CT compared to MRI-CT fusion differed by only 2.4% for the experienced brachytherapist but differed by 4.4% and 9.1% when contoured by experienced radiation oncologists without PB experience. The influence on D90 was 9.3 Gy for the brachytherapists and 14.4 Gy and 30.3 Gy for the other two radiation oncologists.

Others compared prostate volume on TRUS with MRI, without a direct fusion of the two modalities, as in our study. Chung et al. found that TRUS significantly overestimates prostate volume compared to MRI, on average by 15% (SD 25%) [12]. This was even more pronounced in smaller prostates, where it can be as high as 36%.

Some did not find a difference in dosimetry when comparing CT with MRI, although the prostate was much larger on CT than on MRI (ratio MR/CT 0.86 ± 0.14) [13]. The prostate was 16% [14] to 35% [15-16] larger on CT than MRI. The influence of MRI imaging in postimplant dosimetry was studied by Takiar et al. [17]. They found that V100 was about 2% smaller on CT-MRI fusion than on CT alone and D90 was about 9 Gy lower. Both differences were significant. In a similar study by Brown et al., the differences in V100 (7%) and D90 (32Gy) between CT and MRI-CT were even larger [7].

Other disease sites have similar issues in defining the target depending on the imaging modality. A recent comparison of 23 experts in gynecological radiation oncology on three different cases of cervical cancer showed considerable differences, especially for MRI-based contours. The conformity index was higher for CT-based contours. In general, the conformity index between experts ranged from 0.37 to 0.48 and was slightly better for CT. MRI-based contours were consistently smaller than CT contours, but CT contours were more similar [18].

Although this was not evaluated in this present study, we believe that MRI reduces interobserver variability in prostate contouring.

We further believe that every center practicing PB should establish its standard of care by determining if implant quality is well evaluated whether on CT alone or in combination with MRI to be able to compare their results to those in the literature.

## Conclusions

Despite the use of intraoperative TRUS images to determine the prostate volume on day-30 MRI and CT, differences between both modalities remained clinically significant in nearly half the patients. This difference had a direct influence on the value of D90. *’*Even though many advances have been made in prostate imaging in the last few years, the exact determination of prostate contours remains a major challenge and is essential to evaluate implant quality.
